# Unusual association of acquired dermal melanocytosis: A case report

**DOI:** 10.1016/j.jdcr.2026.08.056

**Published:** 2026-09-15

**Authors:** Miguel Gutierrez Jimenez, Luis Valda Rodríguez, Melissa Nicole Quispe, Lizeth Quiroga Conde

**Affiliations:** a Department of Dermatology, Hospital Obrero N° 1, La Paz, Bolivia; b MEDIDERM Dermatology Center, La Paz, Bolivia

**Keywords:** acquired dermal melanocytosis, Hori nevus, Ito nevus, Sun nevus

## Introduction

Dermal melanocytoses are pigmentary disorders characterized by blue-gray macules resulting from proliferation of fusiform and dendritic melanocytes within the dermis. They are classified as congenital or acquired. Congenital forms include Mongolian spot, nevus of Ota, nevus of Ito, and blue nevus, all present at birth or shortly thereafter. Acquired forms (ADM) include Hori nevus, Sun nevus, acquired Ito nevus, and Watanabe infraorbital melanosis.^1^

We present a case of ADM that is remarkable for the simultaneous coexistence of Hori nevus, Sun nevus, and acquired Ito nevus in the same patient, along with concurrent primary biliary cholangitis, cutaneous basal cell carcinoma, and breast carcinoma.

## Case report

A 74-year-old Bolivian woman with no significant past medical history and no Asian ancestry presented with a 4-year history of hyperpigmented patches on the face, chest, and upper extremities. She was initially admitted to the internal medicine service for generalized jaundice secondary to autoimmune cholangitis and was subsequently referred to dermatology for evaluation of her cutaneous lesions.

On examination, blue-gray pigmented patches with irregular borders were noted in the frontotemporal regions, nasal root, infraorbital areas, and nasal alae, consistent with Hori nevus. Isolated, mottled, hyperpigmented macules were present on the zygomatic regions, portions of the cheeks, nasal dorsum, and mid-forehead, consistent with Sun nevus (Fig 1).

**Fig 1:**
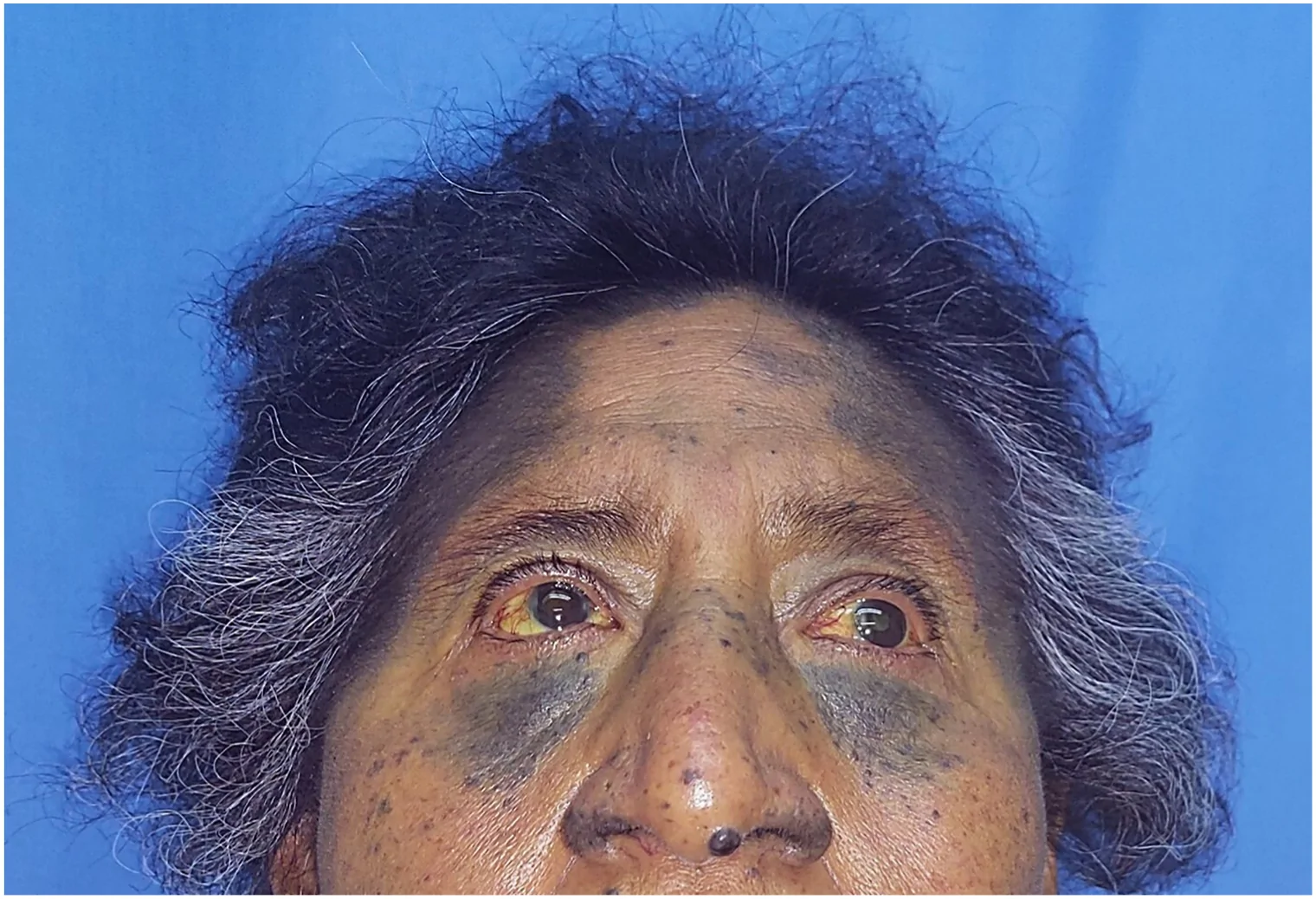
Acquired dermal melanocytosis. Hori and Sun facial nevi. Note icteric conjunctivae and pigmented basal cell carcinoma on the nasal lobule.

Bilateral blue-green patches with irregular borders were observed on the deltoid, scapular, clavicular, and proximal upper extremity regions, consistent with acquired bilateral Ito nevus (Fig 2). Additional blue-green, circumscribed, isolated plaques of varying size were present on the ventral and dorsal thorax (Fig 3).

**Fig 2:**
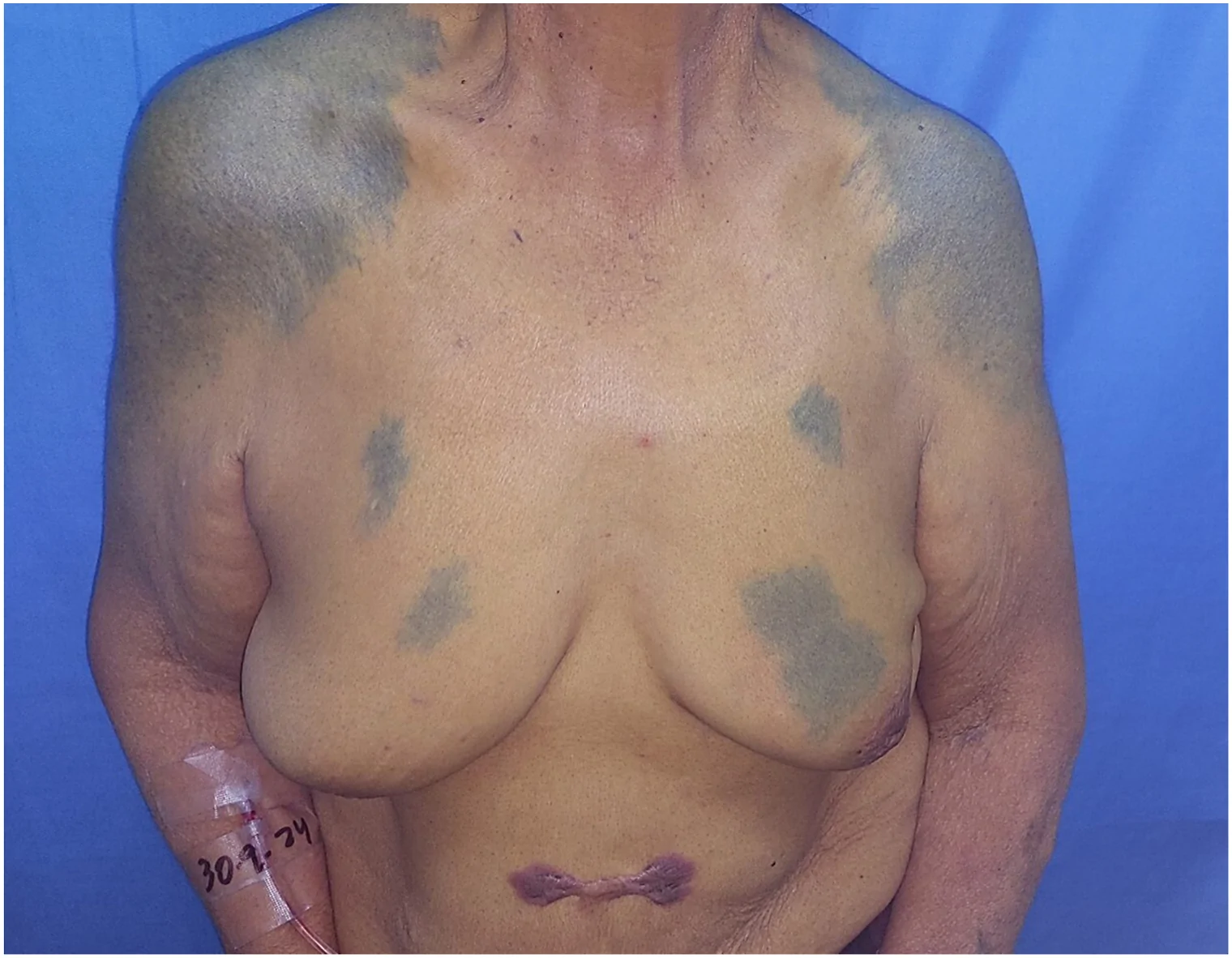
Acquired dermal melanocytosis. Acquired Ito nevus on the arms and anterior chest.

**Fig 3:**
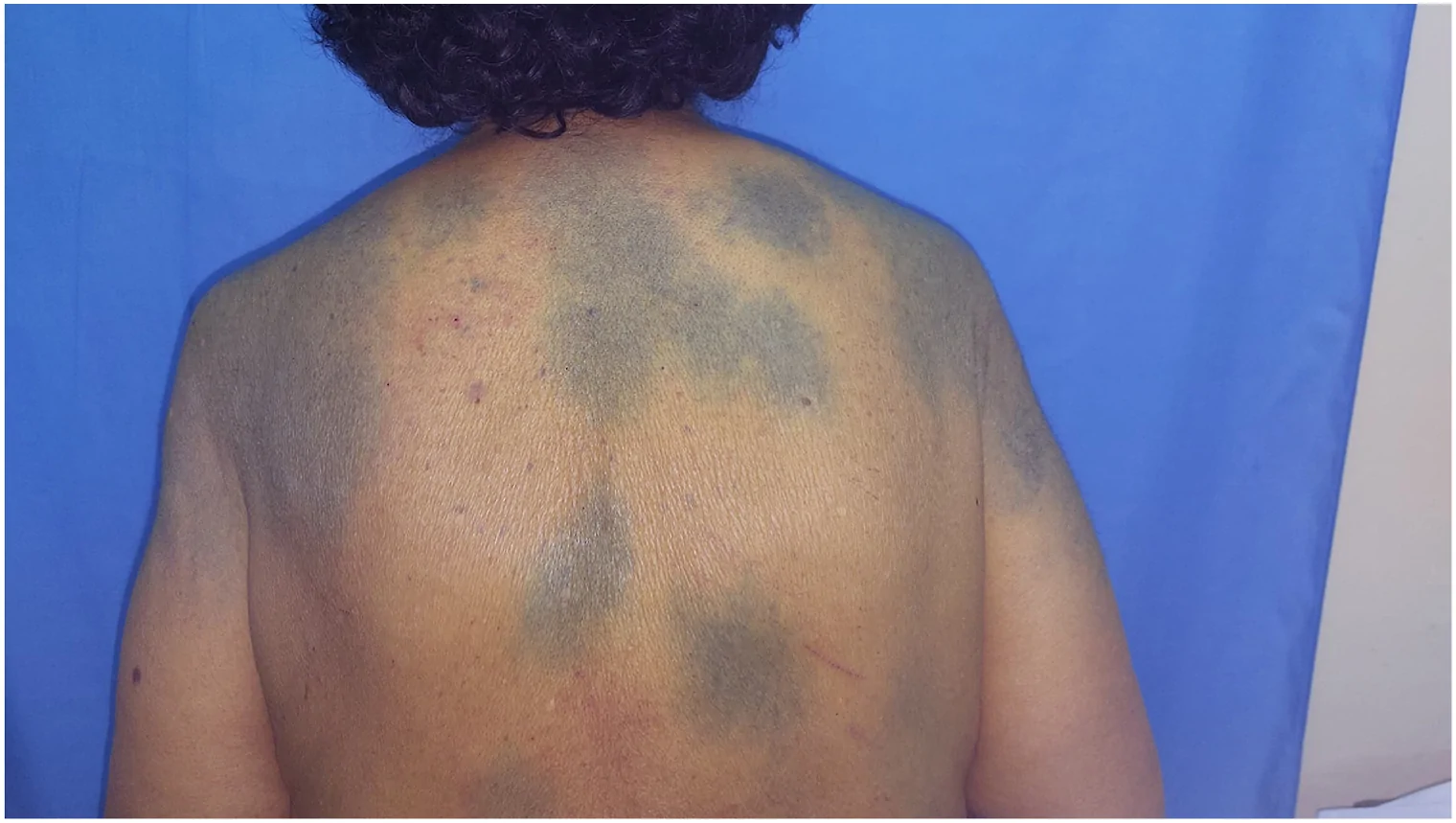
Acquired dermal melanocytosis. Acquired Ito nevus on the scapulae and dorsal thorax.

Given the acquired onset of the pigmented lesions, Nevus of Ota and Nevus of Ito, which are typically congenital conditions, were excluded from the differential diagnosis.

No ocular or mucosal pigmentation was observed.

The patient reported no history of exposure to medications or toxic agents known to induce hyperpigmentation, including arsenic.

Additionally, she had a circumscribed, darkly pigmented nodule on the nasal lobule and a ductal breast nodule with ulceration and skin retraction. Biopsy of the lesions showed a pigmented basal cell carcinoma and breast carcinoma, respectively.

Dermoscopy of the pigmented lesions revealed a homogeneous, mottled blue-gray pattern. Biopsy of a frontal lesion showed a mildly atrophic epidermis with dendritic melanocytes and melanophages distributed among collagen bundles in the reticular dermis (Fig 4).

**Fig 4:**
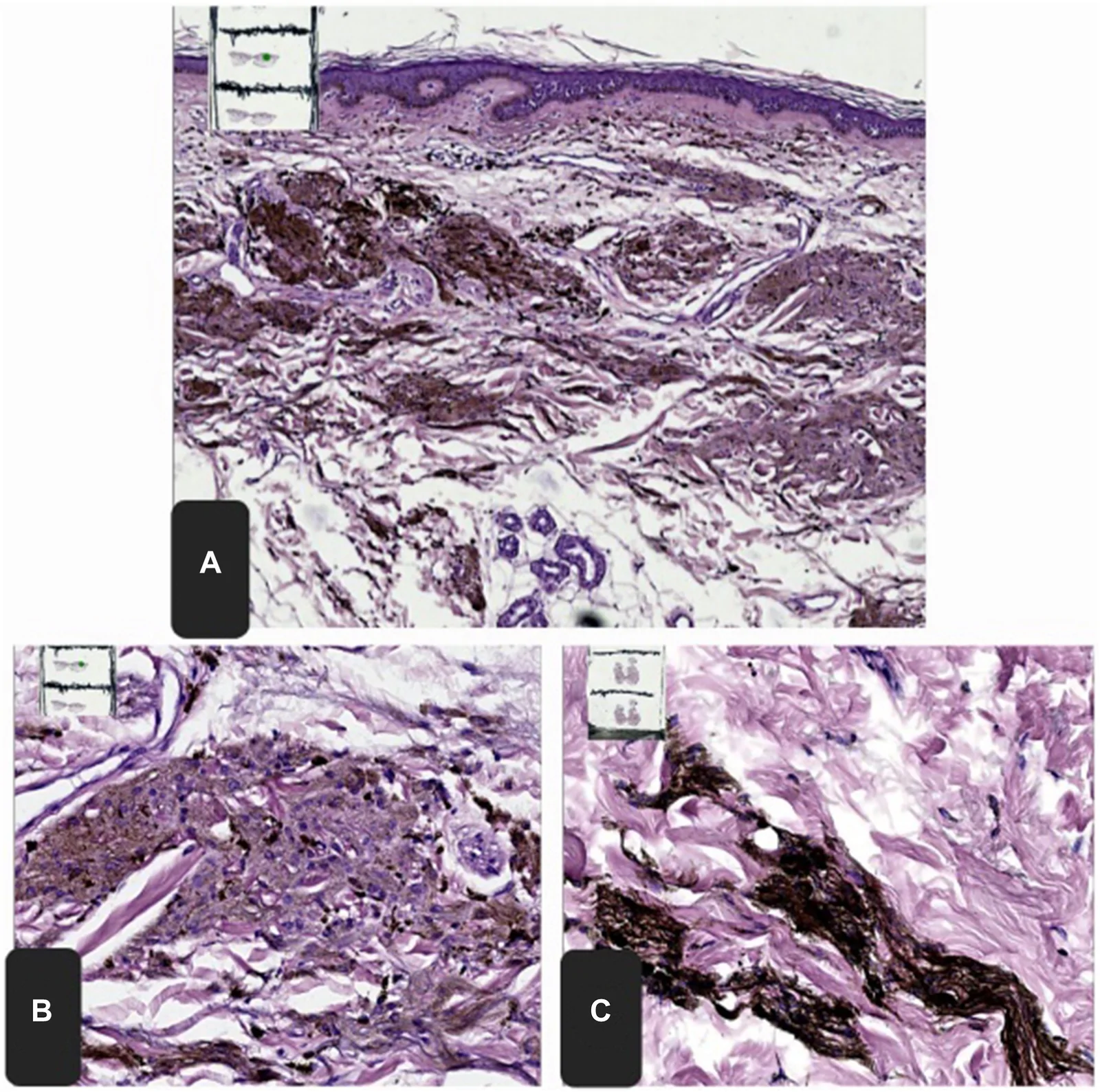
Acquired dermal melanocytosis. Histopathology: **(A)** Proliferation of melanocytes in the reticular dermis; **(B)** Fusiform melanocytes without atypia; **(C)** Melanosis among collagen fibers.

Immunohistochemical and other special stains were not performed due to technical limitations of our hospital's pathology laboratory.

Laboratory evaluation revealed elevated transaminases and bilirubin. Serologies for hepatitis A, B, and C were negative. Anti-mitochondrial and anti-Sp100 antibodies were positive, consistent with primary biliary cholangitis.

Immunosuppressive therapy with prednisone and mycophenolate mofetil improved the jaundice. The patient was transferred to Oncology for management of the breast neoplasm. The basal cell carcinoma was surgically excised. The pigmented nevi did not require treatment.

## Discussion

The first description of ADM is attributed to Mevorah et al,^2^ who in 1977 reported a Portuguese woman with ADM localized to the hand since 11 years of age.

In 1984, Hori et al^3^ described a new entity termed acquired bilateral nevus of Ota-like macules (ABNOM) in 22 Japanese patients presenting with blue-brown macules on the forehead, temples, eyelids, cheeks, and nose. Unlike nevus of Ota, there was no ocular or oral mucosal involvement.

In 1987, Sun et al^4^ studied 110 Chinese patients with acquired, blue-gray, mottled nevi, frequently bilateral, in the zygomatic region, termed naevus fusco-caeruleus zygomaticus. The rounded lesions ranged from 1 to 3 mm in diameter, numbering 3 to 43. This condition is more common in women and differs from ephelides by its color and larger size.

A topographic variant of ADM localized to the upper back was reported in 1991 by Ono et al^5^ in 7 elderly Japanese men. Exclusive localization of ADM to the hands was reported in 1993 by Fukuda et al.^6^

In 2014, Watanabe^7^ described 12 Japanese patients with acquired subpalpebral pigmentation contralateral to a congenital nevus of Ota, termed infraorbital annular melanosis.

In 1954, Ito^8^ reported a Japanese woman with a unilateral congenital pigmented lesion in the acromioclavicular region, anterior chest, and back, designated naevus fusco-ceruleus acromiodeltoideus. Acquired and bilateral forms of Ito nevus have been rarely reported.^9^

The distinction among all ADM subtypes is based on anatomic distribution of the pigmented macules, as histologic findings are similar across subtypes. The association of congenital, bilateral nevi of Ota and Ito has been described in several publications. An acquired Hori nevus associated with a congenital nevus of Ota was reported in a Korean patient.^10^

A thorough literature review did not reveal any previously reported cases of multiple ADM subtypes coexisting in a single patient. Our case is notable for the simultaneous association of several ADM subtypes — Hori nevus, Sun nevus, and acquired Ito nevus — in 1 individual. The concurrent presence of primary biliary cholangitis and carcinomas in this patient is likely coincidental rather than pathogenetically related.

## Conflicts of interest

None disclosed.
